# Supplementary figures and images for: Live Vaccination with Blood-Stage Plasmodium yoelii 17XNL Prevents the Development of Experimental Cerebral Malaria
Source: Vaccines (Basel). 2022 May 11;10(5):762. doi: 10.3390/vaccines10050762 (PMC9145751; doi:10.3390/vaccines10050762)

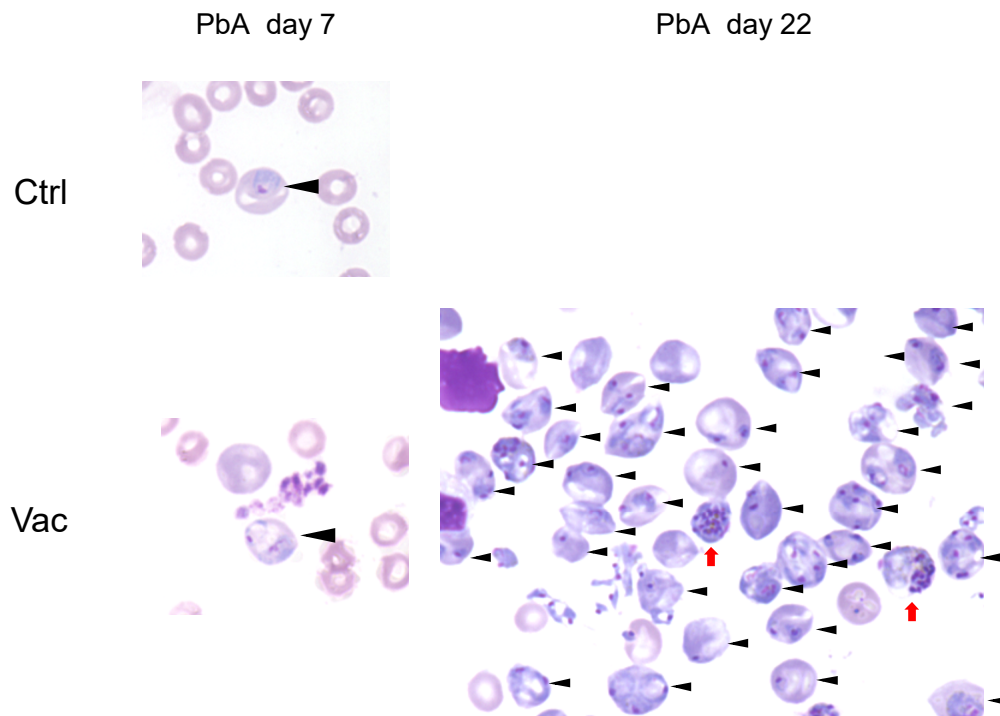

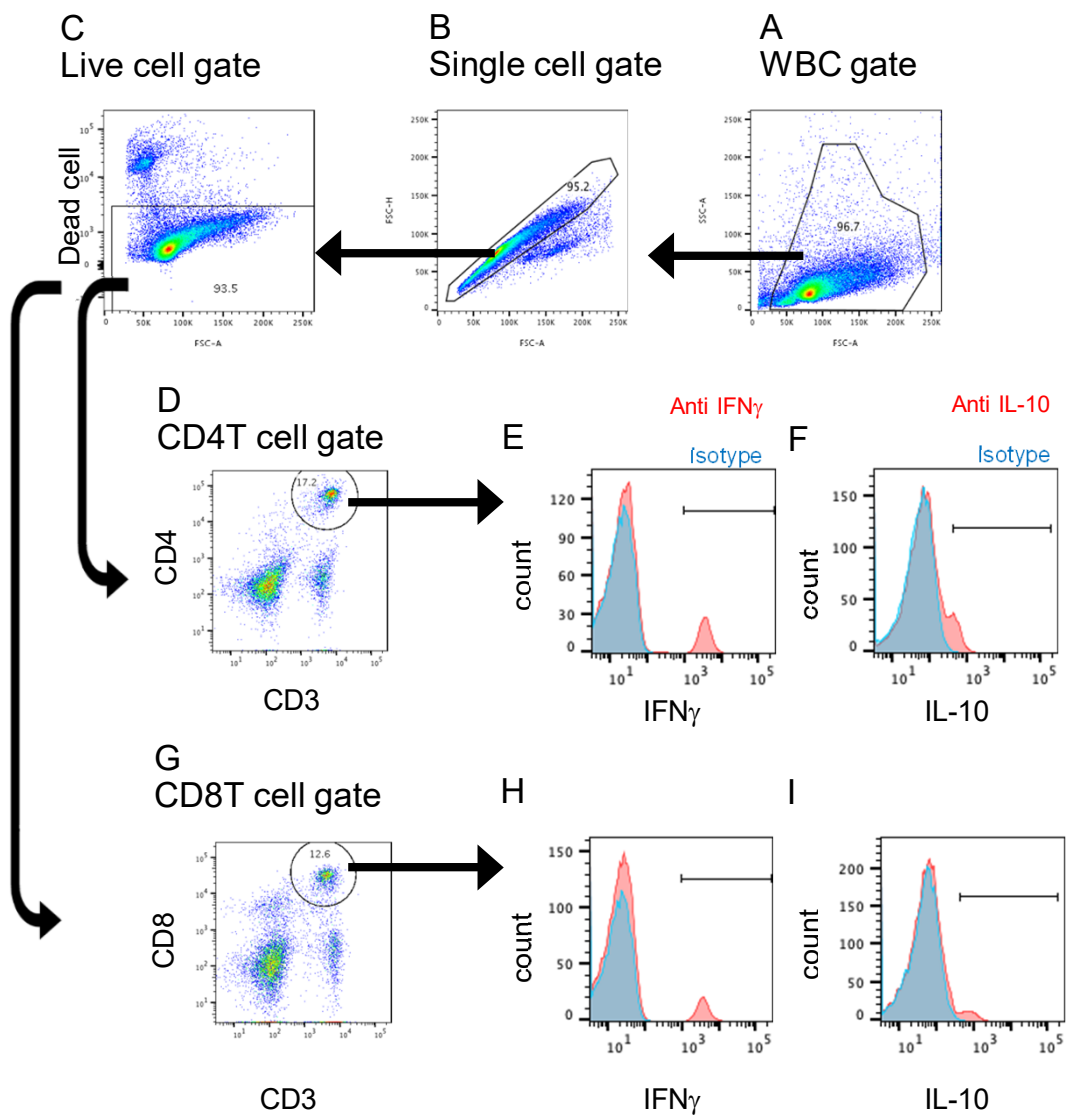

Supplement: Supplementary file 1 [file vaccines-10-00762-s001.zip › vaccines-1661057-SI.pdf]
